# Impact of mono‐ or multitherapy on ocular surface health and quality of life after 5 years of follow‐up in the Glaucoma Intensive Treatment Study (GITS)

**DOI:** 10.1111/aos.70078

**Published:** 2026-01-29

**Authors:** Gauti Jóhannesson, Christina Lindén, Johan Aspberg, Sabina Andersson‐Geimer, Anders Heijl, Boel Bengtsson, Dorothea Peters

**Affiliations:** ^1^ Department of Clinical Sciences, Ophthalmology Umeå University Umeå Sweden; ^2^ Department of Clinical Neuroscience Karolinska Institutet, St Eriks Eye Hospital Stockholm Sweden; ^3^ Department of Ophthalmology University of Iceland Reykjavik Iceland; ^4^ Department of Clinical Sciences in Malmö, Ophthalmology Lund University Malmö Sweden; ^5^ Department of Ophthalmology Skåne University Hospital Malmö Sweden

**Keywords:** glaucoma, ocular surface disease, quality of life, treatment

## Abstract

**Aim:**

To evaluate the impact of initial mono‐ versus multitherapy on the ocular surface and related quality of life after 5 years follow‐up in the Glaucoma Intensive Treatment Study (GITS).

**Method:**

The study included patients with primary open‐angle glaucoma and pseudoexfoliation glaucoma who completed 5‐year follow‐up in GITS. Assessment of ocular surface disease (OSD) symptoms was done using a Swedish Translation of the OSD Index (OSDI). Signs of OSD were assessed with tear break‐up time (BUT), Schirmer I test and staining using Lissamine green. Rasch analysis was used to analyse OSDI results.

**Results:**

Data on OSD symptoms were available at 5 years in 90% (219/242) of all participants initially included in GITS. Subjective or objective OSD findings did not differ significantly between mono‐ and multitherapy. More than 90% of patients in both arms reported no or little subjective ocular surface problems and showed no or minimal staining with Lissamine green at the 60‐month visit. Furthermore, 46% had normal BUT and 60% normal Schirmer tests. Use of preservative‐free drops or need for additive lubricating tear drops did not differ between the arms.

**Conclusion:**

We found no differences in objective or subjective impact on ocular surface between the two randomization arms. However, a subgroup of glaucoma patients had more severe OSD irrespective of the amount of topical glaucoma treatment received, and this should be considered when choosing glaucoma therapy treatment in this subgroup by considering laser treatment or non‐preserved eye drops.

## 1 INTRODUCTION

According to the mission statement of the European Glaucoma Society guidelines for glaucoma, the well‐being and quality of life of glaucoma patients are influenced by not only their visual function but also the side effects of their treatment (EGS, 2025). Thus, it is not sufficient to only focus on lowering the intraocular pressure (IOP) or halting progression if the patient suffers from undesirable side effects (EGS, 2025; Johannesson et al., 2024).

Another important aspect to consider in glaucoma care is the patients' compliance to the prescribed treatment. It is well established (Stalmans et al., 2020) that there is a strong correlation between poor compliance and the severity of side effects due to either the active drug itself or excipients in the eye drops that ensure the effectiveness and stability of the drops as well as preventing microbial growth. A common cause of side effects, apart from the active substance, is preservatives and this has sparked an increased use of preservative‐free alternatives (Baudouin et al., 2010; Scelfo et al., 2023).

The Glaucoma Intensive Treatment Study (GITS) (Bengtsson et al., 2018; Linden et al., 2018) was designed to test the hypothesis if initial multi‐treatment with three IOP‐lowering substances together with laser trabeculoplasty (LTP) was superior to traditional stepwise treatment starting with one IOP‐lowering substance only. The 5‐year results showed that the efficacy of multi‐treatment was highly dependent on initial IOP and showed that multi‐treatment was more beneficial with regards to visual field progression in glaucoma patients with higher untreated IOP (Bengtsson et al., 2024).

Still, the question remains if the positive visual field results of the initial intensive treatment come with a cost of poorer ocular surface environment and quality of life (QOL). The current study tests the hypothesis that initial multitherapy increases the risk of ocular surface‐related complications compared with monotherapy, based on both subjective and objective findings in the 5‐year follow‐up of GITS.

## 2 METHODS

The GITS was a prospective randomized open‐label trial conducted in Malmö and Umeå in Sweden. The study was registered at EudraCT (Ref no.: 2013–002895‐42) and approved by both the Regional Ethical Review Board in Lund, Sweden (Ref. no.: 2013/697) and the Swedish Medical Product Agency (Ref no.: 5.1–2013‐64 667). The GITS adhered to the tenets of the Declaration of Helsinki and all included patients provided written informed consent.

A detailed description of the design of GITS has been published previously (Bengtsson et al., 2018). Briefly, the GITS enrolled 242 patients with newly diagnosed, manifest open‐angle glaucoma (including both primary open‐angle glaucoma and pseudoexfoliation glaucoma). Only patients without any history of intraocular pressure (IOP) lowering therapy before study start were included. Participants were randomized to either initial monotherapy (starting with a single IOP‐lowering drug with a stepwise escalating strategy) or initial multitherapy (starting with three IOP‐lowering drug classes together with a 360° laser trabeculoplasty). During follow‐up, the treating ophthalmologist was free to change therapy if deemed necessary due to visual field progression or unacceptable increased IOP. Furthermore, either preservative‐free or preserved eye drops could be prescribed. Included patients were scheduled for 60 months of follow‐up. Evaluation of patient adherence to the topical treatment was done according to the study protocol described in detail elsewhere (Bengtsson et al., 2018). In short, patients' compliance was assessed at each follow‐up visit using an open‐ended question from the study personnel, unless the patient brought up the topic themselves.

Presence of ocular surface disease (OSD) was evaluated at the last follow‐up visit (60 months). Objective signs of OSD were assessed by one ophthalmologist per patient using three diagnostic tests: (1) break‐up time (BUT) of the tear film, (2) Schirmer I test and (3) staining using Lissamine green. Assessment of OSD signs was performed in both eyes; however, only the results of one eye per patient were included in the present analyses. That eye was the study eye if only one eye was included as the study eye. If both eyes of a patient were included as study eyes, the one with worse objective OSD signs was chosen. In cases where both study eyes presented with the same level of objective OSD signs, one eye was randomly chosen.

BUT was measured in seconds and defined as the time elapsing between the last blink of the patient and the formation of small dry areas on the surface of the cornea. BUT was measured in both eyes one after the other, always starting with the right eye. Fluorescein was applicated onto the outer surface of the eye with a fluorescein ophthalmic strip (I‐Dew Flo, Entod Research Cell Ltd., London, Great Britain). The patient was then instructed to blink a couple of times to ensure the mixing of the fluorescein with the tear film and then asked to keep the eyes open. A BUT of >10 s was deemed normal (level 1). The Nordic guidelines for Dry Eye Disease (Heegaard et al., 2022) were used to define different severity levels of OSD according to the BUT: Level 2—BUT 6–10 s; Level 3—BUT 1–5 s; Level 4—immediate break‐up.

The Schirmer I test was conducted without topical anaesthesia in both eyes simultaneously using calibrated filter strips (I‐DEW Tearstrips, Entod Research Cell Ltd., London, Great Britain). Patients were asked to keep their eyes closed during the test. The strips were removed after 5 minutes, and the amount of wetting of the filter strips was measured in millimetres. A Schirmer score of >10 mm was defined as normal. According to the Nordic guidelines for Dry Eye Disease, a score of 6–10 mm corresponded to severity Level 2, a score of 3–5 mm to severity Level 3 and a score of ≤2 mm to severity Level 4.

Staining was done without topical anaesthesia using a Lissamine Green strip (1‐DEW green, Entod Research Cell Ltd., London, Great Britain). Corneal staining was evaluated with the upper eyelid slightly lifted. Conjunctival staining on the temporal zone was graded while the patient looked nasally, and the nasal zone was graded while the patient looked temporally. Oxford grading scheme was used to grade the staining from grade 0 to grade 5 (Bron et al., 2003) Staining grades were then divided into three severity levels (grade 0 = normal, grade 1 = minimal staining, grade 2 or more = more than minimal staining).

Assessment of subjective symptoms of OSD was done using a Swedish translation of a validated questionnaire, the Allergan Ocular Surface Disease Index (OSDI; Allergan, Inc.) (J, 2004) The OSDI is a self‐administered questionnaire comprising 12 items divided into three subgroups (ocular symptoms, vision‐related function and environmental triggers for OSD). Each item is scored on a five‐category Likert‐type scale ranging from 0 points indicating no problems at any time to 4 points indicating symptoms all the time. The OSDI score is calculated from the three subscale scores taking the number of items answered into account. OSD severity stage is categorized by the sum score: 0–12 points indicate a normal ocular surface, 13–22 points mild OSD, 23–32 points moderate OSD and 33–100 points severe OSD. We calculated and scored the OSDI results according to the published guidelines (J, 2004).

In addition, subjective problems with OSD were determined using item number 4 of the self‐administered National Eye Institute Visual Functioning Questionnaire‐25 (NEI VFQ‐25 Swedish): *‘How much pain or discomfort have you had in and around your eyes (for example, burning, itching, or aching)?’* Answers to item number 4 of the VFQ‐25 from the 1‐month visit and the 60‐month visit were noted. Differences in the grading of ocular surface discomfort between the two administration occasions were calculated as number of steps in either increase or decrease of discomfort.

Only GITS participants with available assessment of OSDI at the 5‐year follow‐up visit were included in the present cohort study.

Usage of contact lenses was noted before enrolment for each patient. Furthermore, prescription of preservative‐free eye drops, changes in therapy from preserved to preservative‐free eye drops due to side effects and prescription of lubricating eye drops during follow‐up was noted.

### 2.1 Statistical analyses

OSDI results were evaluated with Rasch analysis using the Winstep software version 4.7.0.0 (Winsteps, Chicago, IL, USA). However, Rasch analysis of OSDI results revealed an unsatisfactory person separation index of 0.30. Neither did we obtain a satisfactory person separation index when using a four‐category response structure. Instead, proportions and empirically derived 95% confidence intervals (CI) of answers to the 12 OSDI items were calculated separately and compared between the two randomization groups. The calculation of proportions of answers allowed us to compare the two randomization groups for each item separately. However, it did not allow us to grade the subjective OSD‐related symptoms from mild to severe or to rank individual patients according to their subjective OSD symptoms. Potential differences in age were analysed using *t*‐tests, Pearson's chi‐squared tests were used for categorical data. Distribution of answers to the OSDI as well as changes of subjective symptoms over time were compared between the two randomization groups using Mann–Whitney U‐tests. Statistical significance was set to *p* values < 0.05. IBM SPSS version 27.0 was used for statistical analyses.

## 3 RESULTS

**FIGURE 1:**
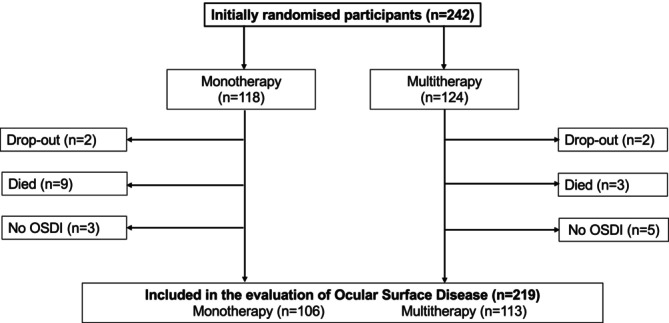
Flowchart illustrating the identification of eligible patients among initially randomized participants in the Glaucoma Intensive Treatment Study (GITS). OSDI, ocular surface disease index.

Data on subjective OSD symptoms from the 60‐month visit were available in 90% (219 of 242) of all participants initially included in the GITS; in 90% (106 of 118) and 91% (113 of 124) randomized to the monotherapy arm and the multitherapy arm, respectively (Figure 1). At the 60‐month visit, 59% (63/106) of patients initially randomized to monotherapy were still treated with a maximum of one, while 86% (97/113) of patients initially randomized to multitherapy were still treated with at least three different IOP‐lowering substances. The two randomization groups did not differ significantly in most clinical characteristics at the 60‐month visit (Table 1). However, significantly more eyes underwent cataract surgery during follow‐up in the multitherapy group compared with the monotherapy group (*p* = 0.023, Table 1). No difference in the prescription of lubricant eye drops during the study or treatment changes due to adverse drug reactions (*p* > 0.05, Table 1) was found. Few participants reported wearing contact lenses before enrolment (nine patients, six in the monotherapy arm and three in the multitherapy arm).

**TABLE 1:** Clinical characteristics of included patients at 60‐month visit: (a) all patients, (b) patients initially randomized to monotherapy, (c) patients initially randomized to multitherapy.

	All patients (*n* = 219)	Initially randomized to monotherapy (*n* = 106)	Initially randomized to multitherapy (*n* = 113)	*p* *
Age at 60‐month visit, years, mean ± SD	72.8 ± 6.5	72.1 ± 7.1	73.6 ± 5.8	0.082
Sex, *n* (%)				0.670
Men	121 (55)	57 (54)	64 (57)	
Women	98 (45)	49 (46)	49 (43)	
Glaucoma type, *n* (%)				0.893
POAG	160 (73)	77 (73)	83 (73.5)	
PEXG	59 (27)	29 (27)	30 (26.5)	
Study eye(s), *n* (%)				0.602
Only one	168 (77)	82 (77)	84 (74)	
Both eyes	51 (23)	24 (23)	29 (26)	
Prescription of lubricant eye drops, *n* (%)				0.094
No	130 (59)	69 (65)	61 (54)	
Yes	89 (41)	37 (35)	52 (46)	
Preservative‐free drugs,^a^ *n* (%)				0.787
No	165 (75)	79 (74.5)	86 (76)	
Yes	54 (25)	27 (25.5)	27 (24)	
Changed treatment due to adverse drug reaction,^a^ *n* (%)				0.357
No	159 (73)	80 (75)	79 (70)	
Yes	60 (27)	26 (25)	34 (30)	
Cataract surgery,^a^ *n* (%)				0.023
No	138 (63)	76 (72)	62 (55)	
Yes, during follow‐up	54 (25)	18 (17)	36 (32)	
Yes, before study start	27 (12)	12 (11)	15 (13)	

Abbreviations: PEXG, pseudoexfoliative glaucoma; POAG, primary open‐angle glaucoma; SD, standard deviation.

^a^ Study eye included into the analyses.

* Pearson's chi‐squared test for all but age (*t*‐test).

### 3.1 Objective OSD signs

**FIGURE 2:**
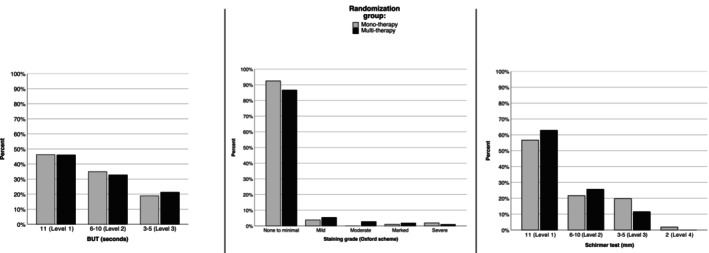
Objective signs of OSD at 60‐month visit presented for the two randomization groups separately. No statistically significant difference in OSD severity level was found between the two randomization groups for BUT time, staining with Lissamine green and Schirmer test. BUT—break‐up time, OSD, ocular surface disease.

Nearly half of the participants (46%, 101 of 219) had a normal BUT, 60% (131 of 219) had normal Schirmer test results and 90% (193 of 214; 2 patients did not want to do the examination, three patients provided no reliable results) showed none to minimal staining with Lissamine green at the 60‐month visit. Results of the three tests are shown separately for the two randomization groups in Figure 2. No statistically significant difference in OSD severity level was found between the two randomization groups for any of these tests (all *p* > 0.05).

### 3.2 Subjective OSD symptoms

**FIGURE 3:**
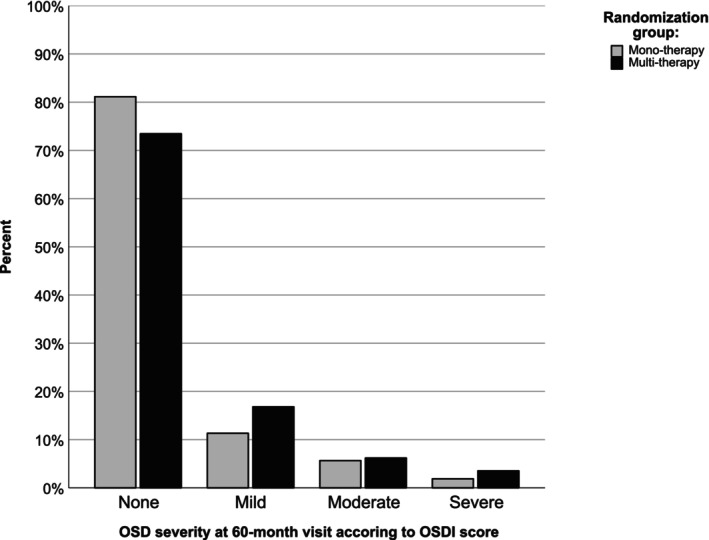
Subjective OSD severity at 60‐month visit according to OSDI sum score presented for the two randomization groups separately. There was no significant difference in the distribution of OSD severity level between the two randomization groups. OSD, ocular surface disease; OSDI, ocular surface disease index.

Most participants rated their OSD symptoms as either not present at any time or as present some of the time (Table 2). Less than 5% of the participants rated their OSD symptoms as present more often (Table 2). No significant differences in the distribution of reported time with OSD symptoms could be found between the two randomization groups (items analysed separately: all *p* > 0.05, Table 2). Furthermore, there was no significant difference in the distribution of OSD severity level (determined from the OSDI sum score) between the two groups (Figure 3, *p* = 0.185).

**TABLE 2:** Results of the Ocular Surface Disease Index (OSDI) presented separately for the two randomization arms: Monotherapy (*n* = 106) and multitherapy (*n* = 113).

Item	N/A	None of the time		Some of the time		Half of the time		Most of the time		All of the time	
	*n* (%)	P (95% CI)		P (95% CI)		P (95% CI)		P (95% CI)		P (95% CI)	
		Mono	Multi	Mono	Multi	Mono	Multi	Mono	Multi	Mono	Multi
Sensitivity to light	0 (0)	0.764 (0.723–0.805)	0.664 (0.602–0.726)	0.179 (0.142–0.216)	0.248 (0.207–0.289)	0.038 (0.019–0.057)	0.044 (0.025–0.063)	0.009 (0.000–0.018)	0.018 (0.006–0.030)	0.009 (0.000–0.018)	0.027 (0.012–0.042)
Gritty	0 (0)	0.689 (0.644–0.734)	0.575 (0.529–0.622)	0.274 (0.231–0.317)	0.336 (0.292–0.380)	0.038 (0.019–0.057)	0.053 (0.032–0.074)	0 (0)	0.027 (0.012–0.042)	0 (0)	0.009 (0.000–0.018)
Pain/sore	0 (0)	0.877 (0.845–0.909)	0.832 (0.797–0.867)	0.104 (0.074–0.134)	0.168 (0.133–0.203)	0.019 (0.006–0.032)	0 (0)	0 (0)	0 (0)	0 (0)	0 (0)
Blurred vision	0 (0)	0.670 (0.624–0.716)	0.575 (0.529–0.622)	0.274 (0.231–0.317)	0.336 (0.292–0.380)	0.009 (0.000–0.018)	0.027 (0.012–0.042)	0.028 (0.012–0.044)	0.044 (0.025–0.063)	0.019 (0.006–0.032)	0.018 (0.006–0.030)
Poor vision	0 (0)	0.783 (0.743–0.823)	0.717 (0.675–0.760)	0.160 (0.124–0.197)	0.230 (0.190–0.270)	0.019 (0.006–0.032)	0.009 (0.000–0.018)	0.009 (0.000–0.018)	0.009 (0.000–0.018)	0.028 (0.012–0.044)	0.035 (0.018–0.052)
Reading	2 (1)	0.783 (0.743–0.823)	0.735 (0.693–0.777)	0.151 (0.116–0.186)	0.177 (0.141–0.213)	0.019 (0.006–0.032)	0.009 (0.000–0.018)	0.038 (0.019–0.057)	0.027 (0.012–0.042)	0.009 (0.000–0.018)	0.035 (0.018–0.052)
Driving at night	***61 (28)*** ^1^	0.425 (0.377–0.473)	0.540 (0.493–0.587)	0.208 (0.169–0.247)	0.071 (0.047–0.095)	0.019 (0.006–0.032)	0.035 (0.018–0.052)	0.047 (0.026–0.068)	0.027 (0.012–0.042)	0.028 (0.012–0.044)	0.044 (0.025–0.063)
Working with computer/ATM	2 (1)	0.906 (0.878–0.934)	0.867 (0.835–0.899)	0.066 (0.042–0.090)	0.115 (0.085–0.145)	0.009 (0.000–0.018)	0 (0)	0.009 (0.000–0.018)	0 (0)	0 (0)	0.009 (0.000–0.018)
Watching TV	3 (1)	0.925 (0.899–0.951)	0.903 (0.875–0.930)	0.028 (0.012–0.044)	0.071 (0.047–0.095)	0 (0)	0.009 (0.000–0.018)	0.009 (0.000–0.018)	0 (0)	0.019 (0.006–0.032)	0.009 (0.000–0.018)
Windy conditions	1 (0.5)	0.689 (0.644–0.734)	0.735 (0.693–0.777)	0.236 (0.195–0.277)	0.168 (0.133–0.203)	0.009 (0.000–0.018)	0.018 (0.006–0.030)	0.047 (0.026–0.068)	0.027 (0.012–0.042)	0.019 (0.006–0.032)	0.044 (0.025–0.063)
Low humidity	***35 (16)*** ^2^	0.755 (0.713–0.797)	0.726 (0.684–0.768)	0.076 (0.050–0.102)	0.071 (0.047–0.095)	0.009 (0.000–0.018)	0.009 (0.000–0.018)	0 (0)	0.027 (0.012–0.042)	0 (0)	0.009 (0.000–0.018)
Air condition	6 (3)	0.840 (0.804–0.876)	0.823 (0.787–0.859)	0.104 (0.074–0.134)	0.115 (0.085–0.145)	0 (0)	0.009 (0.000–0.018)	0.009 (0.000–0.018)	0.018 (0.006–0.030)	0.019 (0.006–0.032)	0.009 (0.000–0.018)

*Note*: Grey shade = less than 5% of the participants in this field. Bold and cursive—indicate high number of N/A.

Abbreviations: N/A, not applicable (question 12 *Air condition*—one participant missed to answer); P, proportion.

^1^ 17 (monotherapy)/18 (multitherapy).

^2^ 29 (monotherapy)/32 (multitherapy).

**FIGURE 4:**
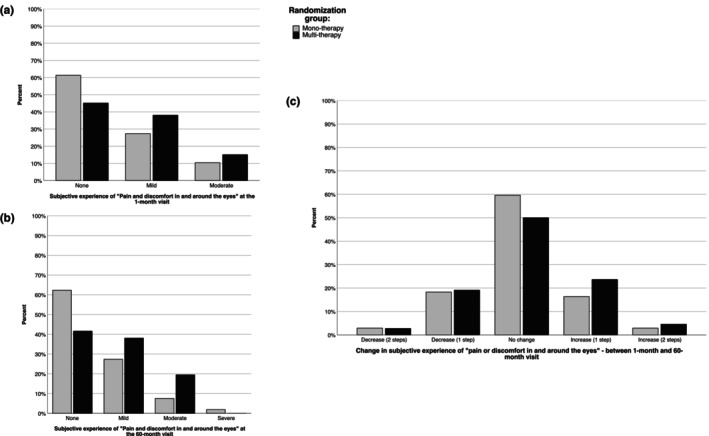
Subjective experience of ‘Pain and discomfort in and around the eyes’ according to item number 4 of the NEI VFQ‐25. Results presented for the two randomization groups separately (a) from the 1‐month visit, (b) from the 60‐month visit and (c) change in subjective experience between 1‐month and 60‐month visit. There was a statistically significant difference between the two randomization groups in the rating of subjective symptoms as ‘*pain or discomfort in and around the eyes’* both at the 1‐month and at the 60‐month visit in all three categories (mild, moderate and severe). However, no significant difference in change of subjective eye symptoms over the 5‐year study period was found between the two randomization groups. NEI VFQ‐25, National Eye Institute Vision Functioning Questionnaire 25.

In 214 participants, results from the VFQ‐25 ‘ocular pain item’ (VFQ‐25, item number 4) were available from both the 1‐month and the 60‐month visit. In total, 54% (116 of 214) did not report any pain or discomfort from the eyes, 34% (72 of 214) rated their eye symptoms as *mild* and 13% (28 of 214) as *moderate* at the 1‐month visit. None of the patients rated their eye symptoms as *severe* or *very severe* at the 1‐month visit (Figure 4). Similar rates were found at the 60‐month visit with only 1% (2 of 214, both randomized to the monotherapy group) of the patients rating their subjective symptoms from the eyes as *severe* (Figure 4).

There was a statistically significant difference between the two randomization groups in the rating of subjective symptoms as ‘*pain or discomfort in and around the eyes’* both at the 1‐month visit (*p* = 0.030) and at the 60‐month visit (*p* = 0.001) (Figure 4). In 55% (117 of 214) of the patients, no change in subjective OSD symptoms from the 1‐month to the 60‐month visit was found; 19% (40 of 214) reported a decrease of symptoms by one step and 3% (6 of 214) by two steps, and 20% (43 of 214) reported an increase of symptoms by one step and 4% (8 of 214) by two steps (Figure 4). Change of subjective eye symptoms did not differ significantly between the two randomization groups (*p* = 0.355).

## 4 DISCUSSION

Overall, in this study on ocular surface health and related quality of life after 5 years of follow‐up in the GITS, there was no statistically significant difference between included patients who had been randomized to receive initial monotherapy or initial multitherapy. There was a trend towards more ‘grittiness’ in the multitherapy group compared with the monotherapy group, with a higher proportion of patients in the monotherapy group stating that they had never experienced grittiness (Table 2). However, given the fact that there was a significant difference in the amount of IOP‐lowering substances between the groups throughout the trial, the similarity in OSD between the two groups as well as the high proportion of generally good ocular surface health in both groups was unexpected.

It is well documented that topical glaucoma medication causes OSD (Stewart et al., 2011), and the prevalence of OSD among glaucoma patients has been reported to affect between 39% and 75% of patients (Asiedu & Abu, 2019). This can be due to the active substance itself, the excipients or preservative agents (Fineide et al., 2022). Cross‐sectional studies (Garcia‐Feijoo & Sampaolesi, 2012; Leung et al., 2008) have shown that a majority of treated glaucoma patients have OSD and a considerable portion of them suffer from moderate or severe OSD. In contrast, in the current study only 23% of the patients had OSD and only 9% experienced more than mild discomfort.

Possible explanations for these non‐conforming results could include differences in OSD definition, dissimilarities in the examined population including sex and age distribution as well as whether the eye drops were preserved or not. With regards to OSD definition, findings in the current study were measured and reported according to international guidelines (Heegaard et al., 2022). However, these do not include assessment of tear break‐up time, and thus, measurement of tear film instability was not included. For assessment of subjective symptoms, we used an internationally validated instrument for ocular surface health, the OSDI (J, 2004), which is the same instrument used in comparative publications (Aapola et al., 2022; Garcia‐Feijoo & Sampaolesi, 2012; Leung et al., 2008). Female sex (Aapola et al., 2022) and increased age (Heegaard et al., 2022) have also been found to be risk factors for dry eye symptoms, but due to randomization these factors did not affect the current results.

Preservatives in eye drops have well‐documented negative effects on ocular surface health causing tear film instability, loss of goblet cells, disruption of corneal epithelium barrier as well as causing damage to deeper ocular tissues (Baudouin et al., 2010). Thus, a higher percentage of preservative‐free medication in one of the groups could contribute to such findings but again, no such difference existed between the groups. Moreover, the correlation between objective and subjective OSD findings is not always obvious (Lee et al., 2020). For instance, Stalmans et al. (2020) found that although nearly half of the examined subjects had OSD, a vast majority of more than 93% were satisfied or very satisfied with their treatment in terms of tolerability. Yet, another explanation for the discrete OSD findings in our study could be that only a few patients (9%, 10/113) in the multi‐treated arm were given aggressive topical medications, such as brimonidine, a drug that has been shown to have a considerable amount of side effects (Krupin et al., 2011). Finally, a recent review on conjunctival goblet cells in glaucoma concluded that there are indications that prostaglandin analogues could be a better choice of IOP‐lowering treatment than other topical anti‐glaucomatous agents when it comes to sparing the goblet cells (Tiedemann et al., 2019). In the current study, all but 19% of the monotherapy patients received prostaglandin analogues as their initial treatment. Thus, it could be argued that this could potentially explain the lack of difference in OSD severity between the two groups.

Randomized clinical trials are the highest level of evidence, but it needs to be considered that participants in such trials cannot be equated with the follow‐up of regular patients in clinical care. In randomized trials, participants are often looked after more, they are examined more extensively and more often, and their visits are on time according to a strict study protocol. Thus, an explanation for the discrepancies in OSD between the current study and studies on regular patients in clinical care might be due to social desirability bias (Edwards, 1957) where patients in randomized trials might want to please the investigators and be less prone to complain. However, this could only be true for the subjective symptoms, and our objective findings of generally good ocular surface health contradict this argument. It is also of interest that in general, the visual field progression rates reported in GITS after 5 years were lower than expected (Bengtsson et al., 2024), indicating that the included glaucoma patients may differ from ordinary glaucoma patients, although we have not found any explanation for this possible observation.

Thus, our results show no difference in OSD signs or symptoms between the randomized groups despite one of the groups using significantly more topical eye drops, which is intriguing. It may indicate that it is not the amount of eye drops that is of most importance, but rather that certain individuals are more sensitive to topical medication and those persons react to the medication irrespective of the amount given. However, this is in contrast to published literature that indicates the contrary—that there is a dose–response relationship where increased OSD is associated with the amount of given drugs (Baudouin et al., 2010; Leung et al., 2008; Parkkari et al., 2022). Some previous studies reported an increase in OSDI score (worse OSD) with increasing time with a glaucoma diagnosis (Erb et al., 2008; Fechtner et al., 2010; Garcia‐Feijoo & Sampaolesi, 2012) with significantly higher OSDI scores in patients with a glaucoma diagnosis of 6 years or more (Garcia‐Feijoo & Sampaolesi, 2012). Therefore, the results might have been different with a longer follow‐up time. A recent study from Finland reported the prevalence of OSD in an elderly Finnish population to be approximately 10–33% depending on the criteria used. This is in line with our findings (Aapola et al., 2022). We observed that there was a high proportion of missing values in some of the questions where the subjects answered ‘not applicable’ or provided no answer at all. This may in retrospect be due to misunderstanding. For instance, in question seven, which regards driving at night, nearly 30% did not take a stand. Some subjects may have thought it regarded driving at night in general while others may have considered it only for the last week prior to filling in the survey.

Rasch analysis revealed poor targeting of the OSDI in our study population. If targeting is inadequate, the survey questions (item difficulty) will not reflect the patient's severity of subjective OSD (participants' ability). The relatively high proportion of patients without any OSD problems in our study cohort could be an explanation for this problem. Furthermore, we found an unsatisfactory person separation index, meaning that it was not possible to divide the study population into different subjective OSD severity groups using Rasch calculated person measures. Dougherty et al. (2011) evaluated the reliability and validity of the OSDI in a group of postmenopausal women previously diagnosed with OSD using Rasch analysis and found a much better person separation index. The fact that all study participants had been diagnosed with OSD prior to study inclusion is most certainly the main reason for the different results of Rasch analysis found in our study compared with that study. Interestingly, also in the study of Dougherty et al., there was some evidence of poor targeting indicating that the items/questions contained in the instrument do not match well the level of OSD in the patient groups studied (Dougherty et al., 2011).

Notably, there was a significant difference in question four of VFQ‐25 regarding pain and discomfort in the eyes between the mono‐ and multitherapy arms both at Month 1 and Month 60 but there was no difference between the arms in terms of change between Month 1 and Month 60. This may indicate that either there was a difference between the two arms from the start of the trial or that there is a smaller subpopulation that reacts to multitherapy. Given the lack of change over time, the results suggest that patients that do not tolerate multitherapy will show symptoms already from the beginning, enabling the physician to identify and adjust the therapy while the patients who tolerate it from the beginning will most likely do so for years to come.

This study has several strengths and weaknesses. The main strength of the study is its prospective design and tightly controlled execution with good adherence to prescribed therapy and high participation rate throughout the follow‐up period of 5 years. Furthermore, the randomization of the subjects increased the likelihood that the presence of pre‐existing OSD before inclusion in the trial should have been equally distributed in the two randomization arms. The main weakness is the lack of documentation of OSD status at inclusion before any treatment was given. Such documentation would have given valuable information about the baseline status of the participants and would have allowed for a better comparison with the 5‐year follow‐up data. However, due to the study design as a large randomized controlled trial where all patients were randomized into either mono‐ or multitherapy, there is no reason to suspect that the groups differed at baseline.

In conclusion, we found no differences in objective or subjective impact on ocular surface between the two randomization arms at 5 years of follow‐up in GITS. The results showed generally less OSD compared with published literature on glaucoma patients and, surprisingly, contradict the hypothesis that more topical medication results in more OSD. However, a subgroup of glaucoma patients had more severe OSD irrespective of the amount of topical glaucoma treatment received and this should be considered when choosing glaucoma therapy treatment in this subgroup by considering laser treatment or non‐preserved eye drops.

## FUNDING INFORMATION

Financial support was provided through regional agreements between Lund University and Skåne Regional Council (A.L.F.), and between Umeå University and Västerbotten County Council, and also by grants from the Swedish Society for medical research, Knut and Alice Wallenbergs foundation, Cronqvist foundation, Ögonfonden, Swedish medical society foundation, Foundation for visually impaired in former Malmöhus county, King Gustav V and Queen Victoria's freemason foundation, foundations and donations administered by Skåne University Hospital, Crown Princess Margareta's foundation, Margit and Kjell Stolz Foundation, Herman Järnhardt foundation, Ingrid Nordmark's foundation, Insamlingsstiftelserna vid Umeå universitet. None of the funders had any role in the design, conduct, data collection, analysis, interpretation of results or the reporting of the study.

## CONFLICT OF INTEREST STATEMENT

G.J. has received consultant and speaker honoraria from AbbVie, Santen, and Théa. D.P.P. has received speaker honoraria from Santen Pharma AB, Théa Nordic, and Low Vision International, and has served as a consultant for Santen Pharma AB and AbbVie. C.L., B.B., A.H., J.A. and S.A‐G. have nothing to disclose.

## Supporting information


**Table S1.** Treatment in the eye with the highest number of intraocular pressure (IOP) lowering substances at the 60‐month visit in the mono‐ and multitherapy group.
