# Supplementary material for: Impact of mono‐ or multitherapy on ocular surface health and quality of life after 5 years of follow‐up in the Glaucoma Intensive Treatment Study (GITS)
Source: Acta Ophthalmol. 2026 Jan 29;104(5):535–43. doi: 10.1111/aos.70078 (PMC13353630; doi:10.1111/aos.70078)
Supplement: Supplementary file 1 — Table S1. Treatment in the eye with the highest number of intraocular pressure (IOP) lowering substances at the 60‐month visit in the mono‐ and multitherapy group. [file AOS-104-535-s001.docx]

Table S1. Treatment in the eye with the highest number of intraocular pressure (IOP) lowering substances at the 60-months visit in the mono- and multitherapy group.

| **Number of IOP-lowering substances** | **Mono**  **N= 106** | **Multi**  **N= 113** |
| --- | --- | --- |
| None in either eye | 2 (2%) | 1 (1%) |
| **One IOP-lowering substance** |  | |
| - Pg - B - CAI | 61 (58%)  45  16  0 | 7 (6%)  3  3  1 |
| **Two or more pressure lowering substances** |  | |
| **2**   - Pg + B - B + CAI - CAI + Pg | 29 (27%)  25  1  3 | 10 (9%)  8  1  1 |
| **3**   - Pg + B + CAI - Pg + CAI + Symp - Pg + B+ Symp | 13 (12%)  13*  0  0 | 93 (82%)  87  4  2 |
| **4**   - Pg + B + CAI + Symp | 1 (1%) | 2 (2%) |

* = one patient with parasympatomimetic eye drop in the other eye, all other patients with same subset of medication in the other eye

Pg = Prostaglandins (eye drops)

B = Beta-blocker eye drops

CAI = Carbonic Anhydrase Inhibitor eye drops

Symp = Sympatomimetic eye drops

Parasymp = Parasympatomimetic eye drops
